# A systematic analysis on prevalence and sub-regional distribution of undiagnosed diabetes mellitus among adults in African countries

**DOI:** 10.1007/s40200-020-00635-9

**Published:** 2020-09-22

**Authors:** Getenet Dessie, Henok Mulugeta, Desalegne Amare, Ayenew Negesse, Fasil Wagnew, Temsgen Getaneh, Akililu Endalamew, Yibeltal Wubale Adamu, Gizachew Tadesse, Aster Workineh, Sarah Lebu

**Affiliations:** 1 Department of Nursing, School of Health Science, College of Medicine and Health Science, Bahr Dar University, Bahr Dar, Ethiopia; 2 Department of Nursing, College of Health Science, Debre Markos University, Debre Markos, Ethiopia; 3 Department of Human Nutrition and Food Science, College of Health Science, Debre Markos University, Debre Markos, Ethiopia; 4 Department of Midwifery, College of Health Science, Debre Markos University, Debre Markos, Ethiopia; 5 Department of Biomedical Science, College of Medicine and Health Science, Bahr Dar University, Bahr Dar, Ethiopia; 6 Department of Biostatics and Epidemiology, School of public health, College of Medicine and Health Science, Bahr Dar University, Bahr Dar, Ethiopia; 7 School of Public Health, University of California, Berkeley, Berkeley, CA USA

**Keywords:** Diabetes mellitus, Undiagnosed, Meta-analysis, Africa

## Abstract

**Background:**

Despite the high prevalence of diabetes in Africa, the extent of undiagnosed diabetes in the region is still poorly understood. This systematic review and meta-analysis was designed to determine the pooled prevalence of undiagnosed diabetes mellitus among adults in Africa.

**Methods:**

We conducted a systematic desk review and electronic web-based search of PubMed, Google Scholar, EMBASE, and the World Health Organization’s Hinari portal (which includes the SCOPUS, African Index Medicus, and African Journals Online databases), identifying peer-reviewed research studies on the prevalence of undiagnosed diabetes among adult individuals using pre-defined quality and inclusion criteria. We ran our search from June 1, 2018 to Jun 14, 2020. We extracted relevant data and presented descriptive summaries of the studies in tabular form. The I^2^ test was used to assess heterogeneity across studies. A random effects model was used to estimate the pooled prevalence of undiagnosed diabetes mellitus at a 95% confidence interval (CI). Funnel plot asymmetry and Egger’s tests were used to check for publication bias. The final effect size was determined by applying a trim and fill analysis in a random-effects model.

**Results:**

Our search identified 1442 studies amongst which 23 articles were eligible for inclusion in the final meta-analysis. The average pooled prevalence of undiagnosed diabetes mellitus among adults was 3.85 (95% CI: 3.10–4.60). The pooled prevalence of undiagnosed diabetes mellitus based on geographic location was 4.43 (95% CI: 3.12–5.74) in Eastern Africa; 4.72 (95% CI: 2.64–6.80) in Western Africa; 4.27 (95% CI: 1.77–6.76) in Northern Africa and 1.46 (95%CI: 0.57–2.34) in southern Africa respectively.

**Conclusion:**

Our findings indicate a high prevalence of undiagnosed diabetes in Africa and suggest that it may be more prevalent in Western Africa than the rest of the regions. Given the high levels of undiagnosed diabetes in the Africa region, more attention should be paid to incorporating diabetes screening and treatment services into existing diabetes related programs to reduce the prevalence of undiagnosed cases.

**Electronic supplementary material:**

The online version of this article (10.1007/s40200-020-00635-9) contains supplementary material, which is available to authorized users.

## Introduction

Diabetes mellitus (DM) is a broad term used to describe chronic metabolic disorders leading to prolonged hyperglycemia. It is generally classified into 2 main types depending on disease development mechanism [1]. Type 1 DM is not fully understood but is generally due to environmental and genetic factors triggering an autoimmune destruction of β-cells that leads to absolute insulin deficiency. It usually develops during childhood and adolescence. There is limited data available on the incidence or prevalence of Type 1 DM in many LMICs but is generally less common than the second type. Type 2 DM is characterized by insufficient insulin production as well as insulin resistance with the body unable to effectively use the insulin it produces. It is normally diagnosed after the fourth decade of life due to its slow progression and accounts for 90% of all diabetes worldwide [1, 2]. Although it is usually associated with older adults, it has been increasingly reported in children and adolescents. Risks for Type 2 DM include unhealthy diet, obesity, and physical inactivity which have increasingly become more prominent in many LMICs due to the notable changes in diet and lifestyle following urbanization and industrialization [3].

If diabetes is not detected in time for successful management, harmful complications and premature death can follow. Diabetes can damage the heart, blood vessels, eyes, kidneys and nerves, and increase the risk of heart disease and stroke. The International Diabetes Federation (IDF) periodically updates its Diabetes Atlas report which is one of the main global references of diabetes prevalence. The eighth edition of the Diabetes Atlas report estimates that 77% of all diabetes related deaths worldwide occur in Sub-Saharan Africa in people under 60 years of age [4]. The considerable amount of mortality reflects on the ill-equipped healthcare infrastructures which has been unable to properly respond to the burden of the disease. It is evident that Diabetes has become a hidden epidemic in the continent, and it is estimated to worsen. According to the IDF estimation, prevalence of diabetes upsurge by 156% in Africa, 16% Europe, 35% North America and Caribbean and North America and Caribbean 84% in South East Asia by the year 2045 [4]. The report emphasized the high degree of uncertainty in its prevalence estimates due to the lack good quality and up-to-date evidence from sub-Saharan Africa. It stated that over three quarters of the region’s countries and territories lacked primary data on diabetes prevalence in adults. Ethiopia, South Africa and Democratic Republic of Congo are the region’s most populous countries and also have the highest numbers of people with diabetes [5]. However, data sources used for prevalence estimation in these countries were of low quality and limited in number. A large portion of North African countries had a range of low to high quality data. The report further provided a high estimate of undiagnosed DM in sub-Saharan Africa [4].

Despite these estimates, the need for improved diabetes diagnosis and care in sub-Saharan Africa remains unmet with continued low prioritization of screening, research, and prevention [18].

Although it has its limitations, the IDF estimates certainly reflect a true rise in the prevalence of the disease. Additional studies have highlighted the increasing burden of DM by analyzing trends in large scale pooled population-based studies from 1980 to 2014 in African countries and worldwide. The 2016 study which assessed trends of DM concluded that prevalence and number of adults affected by DM has increased faster in low-income and middle-income countries [6]. The results indicated that estimates in Northern Africa (driven by Egypt) and in Southern Africa (driven by South Africa) appeared higher than the global average, whereas estimates for other regions were mostly lower [7]. This shows the lack of up-to-date estimates of DM prevalence in African countries.

On the other hand, there number of country-level reviews and meta-analyses on DM prevalence [8–12]. Although these publications provide locally relevant findings, they are geographically fragmented. In addition, in response to the rapidly growing prevalence of diabetes and the apparent gap in knowledge of true disease impact, a number of primary studies which aim to provide a quantitative evidence of DM prevalence have since been published in countries with high estimates of DM prevalence. With the most recent IDF Diabetes Atlas report published in 2017, there is a lack of updated analysis of national and regional estimates of DM prevalence in African countries [4, 7, 13, 14]. A study published in 2019 utilized these recent primary studies to assess pooled prevalence of undiagnosed DM (UDM) in the African continent and compared variabilities in rural and urban areas to countries such as China, India, Russia, and the USA. The study also concluded that UDM was two times higher in urban populations than in the rural population in African countries [15].

The percentage of UDM is an important public health indicator of the adequacy of current response of diabetes screening and diagnostic measures of local health systems. In addition, since diabetic complications are the cause of the morbidity and mortality associated with the disease, it is urgent that we fill the knowledge gap of both locally relevant country level and sub-regional quantitative evidence of UDM prevalence for targeted interventions. Ultimately, this systematic review and meta-analysis synthesize available evidence to address the lack of up-to-date, nationally representative, and high-quality data that will help to inform policy makers to prioritize implementation of preventative and intervening strategies to reduce the considerable amount of mortality and morbidity posed by DM in African countries. The aim of this review was to determine the pooled prevalence of undiagnosed diabetes among adults in Africa.

## Methods

### Search approach and appraisal of studies

Articles reviewed in this meta-analysis were accessed through electronic web-based database searches, desk reviews of grey literature, and reference list reviews. It is in accordance with the Preferred Reporting Items of Systematic Reviews and Meta-Analysis protocols (PRISMA-P) checklist guidelines [16]. This study was not pre-registered. The electronic databases searched were PubMed, Google Scholar, Embase, and World Health Organization (WHO) database portal for LMICs that includes the Web of Science, SCOPUS, African Index Medicus (AIM), Cumulative Index to Nursing and Allied Health Literature (CINAHL), WHO’s Institutional Repository for Information Sharing (IRIS) and African Journals Online databases. In addition, the researchers found related articles through a desk review of the grey literature available on local shelves and from reviewing the reference lists of already identified journal articles. The authors used the following key terms for the database searches: “prevalence” AND “undiagnosed” AND “diabetes mellitus” combined with names of the 54 African countries. Additional search terms included “diabetes” and “mellitus” OR “diabetes mellitus” AND “adult” AND “population”. These search terms were pre-defined to allow a comprehensive search strategy that included all fields within records and Medical Subject Headings (MeSH terms) (See S1 table). This study also used Boolean operator (within each axis we combined keywords with the “OR” operator and we then linked the search strategies for the two axes with the “AND” operator) to search undiagnosed DM specifically for each African country. Searches were conducted from June 1, 2018 to Jun 14, 2020.

### Inclusion and exclusion criteria

The inclusion criteria used was ‘all English-language, full-text articles on lab-based cross-sectional studies conducted in the Africa region from 2007 to 2020’. In studies published in peer-reviewed journals or found from grey literature, only those conducted by using internationally accepted diagnostic material to measure blood glucose level and whose diabetes mellitus definition criteria was according to internationally accepted definition were included. Studies which reported the prevalence of diabetes mellitus in the full article were eligible for inclusion for this systematic review and meta-analysis. Studies with no accessible full text after using all the PRISMA-P searching strategies and studies which did not report specific outcomes for undiagnosed DM quantitatively were excluded from this systematic review and meta-analysis.

### Data abstraction procedure

The authors used two stages of screening. Primarily, we screened the titles and abstracts based on the criteria set in the protocol. Secondly, we identified potentially relevant articles using titles and abstracts for further re-screening of its full article document. The relevance of the articles was evaluated based on their topic, objectives, and methodology as listed in the abstract. The abstracts were also assessed for agreement with the inclusion criteria. When it was unclear whether an abstract was relevant, it was included for retrieval. At this stage articles deemed irrelevant or out of the scope of the study were excluded and the full text of the write-up downloaded for a detailed review.

### Quality appraisal of individual studies

The Database search results were combined, and duplicate articles were removed manually using Endnote (version X7). The Newcastle-Ottawa Scale (NOS) criteria was used for quality assessment before analysis [17]. Two independent reviewers critically appraised each paper. Disagreements between those reviewers were solved by discussion. If not, a third reviewer was involved to resolve the inconsistencies between the two independent reviewers. The average of the two independent reviewers’ scores was used to determine whether the articles should be included. Articles with NOS quality score of less than six, methodological flaws, incomplete reporting of results, or for which full text was not available were excluded from the final analysis. Study researchers made two separate attempts to contact article authors whenever additional study information was needed; for example, when patient outcome data were incomplete. Risk of bias in the studies was evaluated by using the 10-item rating scale developed Hoy et al. for prevalence studies (see S2 Table) [18].

### Outcome measurement

It was measured as the number of observed glucose level reading above the cut point of WHO definition divided by the number of all adult population in a study multiplied by 100. This gold standard criterion uses fasting plasma glucose ≥7.0 mmol/l (126 mg/dl) or 2–h plasma glucose ≥11.1 mmol/l (200 mg/dl) [19].

### Data analysis

**Fig. 1:**
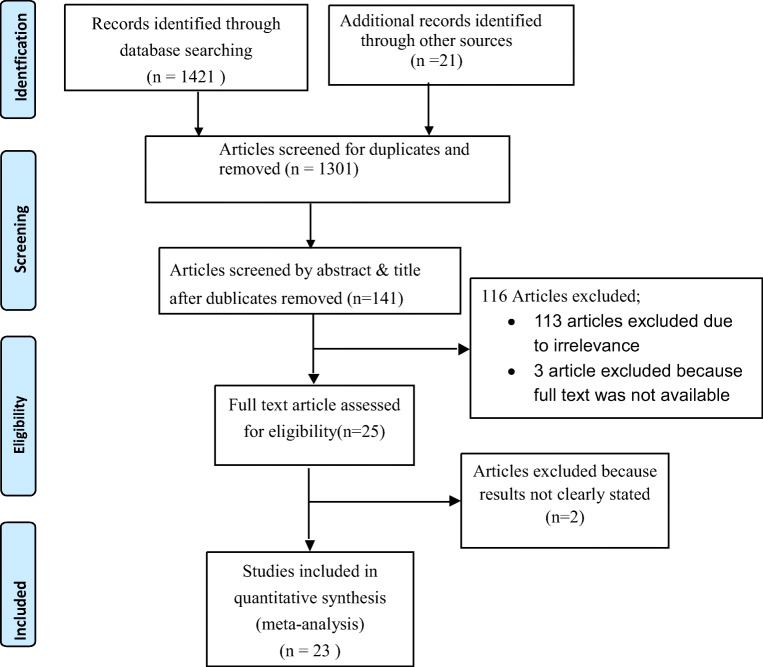
PRISMA-P flow diagram showing the procedure of selecting studies for meta-analysis, 2007–2020, Africa

Information on the study characteristics (time frame, study location, study design, sample size, method of diagnosis, number of undiagnosed diabetes, and age-range of patients) was extracted from each study using a Microsoft Excel spreadsheet template. These data were then transferred to Stata version 14 software to describe the pooled prevalence of undiagnosed diabetes. Heterogeneity across studies was assessed using the inverse variance (I^2^) and Cochran Q statistics with 25% as low, 50% as moderate, and 75% as severe heterogeneity [20]. Since the test statistic indicated significant heterogeneity among studies (I^2^ > 70%, *p* < 0.05), a random effects model was used to estimate the pooled prevalence of undiagnosed diabetes at a 95% confidence interval (CI) and a geographic subgroup analysis was conducted. We used funnel plot asymmetry (Fig. 1) and Egger’s and Begg-Mazumdar Rank correlation tests to check for publication bias [21]. When the results of these tests provided significant evidence of publication bias, the final effect size was determined by applying trim and fill analysis in the random-effects model [22]. To confirm results, two researchers independently carried out the main statistical analysis and results were verified for consistency.

## Results

### Identification and description of studies

The database search and desk review yielded a total of 1442 articles (Fig. 1). Of these, 1421 articles were found in PubMed, Hinari and Google Scholar and other electronic sources. The remaining 21 were found from desk review. After reviewing the titles and abstracts, we excluded 1301 articles due to duplication. One hundred thirteen articles were also excluded with a reason of irrelevance and three additional articles were excluded because the articles were not available in full text [23–25] (Fig. 1). The remaining 25 articles were further assessed for quality and relevance. Two article were excluded due to the lack of clarity regarding outcomes [26, 27]. The remaining 23 studies were included in the analysis (Fig. 1). Of the 23 articles reported, 11 were from Ethiopia [28–38], Three from Ghana [39, 40], and the remaining eight studies from Sudan [41], Benin [42], Botswana [43], Egypt [44], Burkina Faso [45], Zambia [46],Tunisia [47] and South Africa [46].

### Characteristics of included studies

Twenty studies with a total sample of 84, 294 adults were assessed (Table 1). All the studies were lab-based cross-sectional studies reported in peer-reviewed journals and conducted in the region of Africa. A large portion of the studies analyzed were conducted in Ethiopia. Majority of the studies used standardized blood glucose measurement tools and defined the outcome variable based on World Health Organization (WHO) diagnostic criteria, three of the studies did not state the blood glucose level measurement method used. To measure blood glucose level, seven measuring tools; Glucose oxidase method, HemoCue Glucose 201+ apparatus, ONETOUCH Ultra Easy blood glucose meter, AccuCheck Active®, Roche Diagnostic, HumaStar 80 chemistry analyzer, High performance liquid chromatography (HPLC) assay method, and Optium Xceed point-of-care glucometer were used. All of the studies had a large sample size except two studies which had less than 300 participants [40, 43]. Reported response rates were high (>90%), but five of the studies did not report a response rate.

**Table 1:** Characteristics of studies included for systematic review and meta-analysis, 2007–2020, Africa

Authors name	Publication Year	Source Type	Country	Diagnostic criteria	Diagnosis Method	Sample size	Response rate (%)	No of people with outcome	Prevalence (%)	Quality Score
Bouguerra, R., et al. [47]	2007	J	Tunisia (N)	ADA	Glucose oxidase-6 Phosphate Dehydrogenase Method	3729	85.1	277	7.4	7.0
Megerssa YC, et al. [31]	2013	J	Ethiopia (E)	WHO	Humastar 80 Chemistry Analyzer	422	100.0	21	5.0	7.0
Abebe et al. [28]	2014	J	Ethiopia (E)	IDF and WHO	***	1050	97.0	34	3.2	7.0
Sagna Y.et al. [45]	2014	J	Burkina Faso (W)	WHO	Glucometer One Touch Ultra	467	***	15	3.2	7.0
Seifu W.et al. [38]	2015	J	Ethiopia (E)	WHO	***	4371	97.8	55	3.8	7.0
Noor et al. [41]	2015	J	Sudan (N)	ADA	Accu-Check Active®, Roche Diagnostic	1111	100.0	29	2.6	7.0
Djrolo, F. et al. [42]	2015	J	Benin (W)	WHO	Glucometer One Touch Ultra	4597	100.0	361	7.9	7.0
Bailey SL.et al. [46]	2016	J	Zambia (S)	IDF	Optium Xceed Point-of-Care Glucometer	45,767	***	458	1.0	6.5
Bailey SL.et al. [46]	2016	J	South Africa (S)	IDF	Optium Xceed Point-of-Care Glucometer	12,496	***	150	1.2	7.0
Birhanu S.et al. [30]	2016	J	Ethiopia (E)	ADA	Huma Star 80 Chemistry Analyzer	402	97.1	23	5.7	7.0
Zahran AM et al. [44]	2016	J	Egypt (N)	ADA	***	1255	100.0	53	4.2	7.0
Bernard Omech et al. [43]	2016	J	Botswana (S)	ADA	Liquid Chromatography (HPLC) Assay Method	291	***	42	14.4	7.0
Worede et al. [34]	2017	J	Ethiopia (E)	ADA	Glucose Oxidase Method	392	100.0	9	2.3	7.0
Abebe SM.et al. [28]	2014	J	Ethiopia (E)	WHO& IDA	***	2150	97.30%	53	2.5	7.0
Elvis Tarkang et al. [40]	2017	J	Ghana (W)	WHO	ONETOUCH Ultra Easy Blood Glucose Meter	264	100.0	6	2.4	7.0
Elvis Tarkang et al. [48]	2017	J	Ghana (W)	WHO	ONETOUCH Ultra Easy Blood Glucose Meter	387	***	28	7.1	7.0
A.T. Wondemagegn et al. [33]	2017	J	Ethiopia (E)	WHO	Glucose Oxidase-6 Phosphate Dehydrogenase Method	757	95.4	83	11.5	7.0
Kweku et al. [39]	2017	J	Ghana (W)	WHO	ONETOUCH Ultra Easy Blood Glucose Meter	628	100.0	35	5.6	7.0
Wondemagegn et al. [32]	2017	J	Ethiopia (E)	WHO	Glucose Oxidase-6 Phosphate Dehydrogenase Method	530	98.0	46	8.7	6.5
Animaw W, Seyoum Y [29]	2017	J	Ethiopia (E)	WHO	Glucometer One Touch Ultra	1405	95.5	35	2.5	6.5
Bantie, G. M et al. [35]	2019	J	Ethiopia (E)	WHO	Glucose oxidase-6 phosphate dehydrogenase	607	100%	62	10.2	7
Endris T et al. [36]	2019	J	Ethiopia (E)	WHO	Glucose oxidase-6 phosphate dehydrogenase	587	98.20%	29	4.94	7
Dereje N, et al. [37]	2020	J	Ethiopia (E)	WHO	Glucose oxidase-6 phosphate dehydrogenase	627	99	15	2.4	7

*J*, Journal; *E*, East; *W*, West; *N*, North; *S*, South; *IDF*, International Diabetic Federation; *WHO*, World Health Organization Criteria; *ADA*, American Diabetes Association Criteria; *CDC*, Center for Infectious Diseases Criteria

***- Missing information

### Publication Bias

**Fig. 2:**
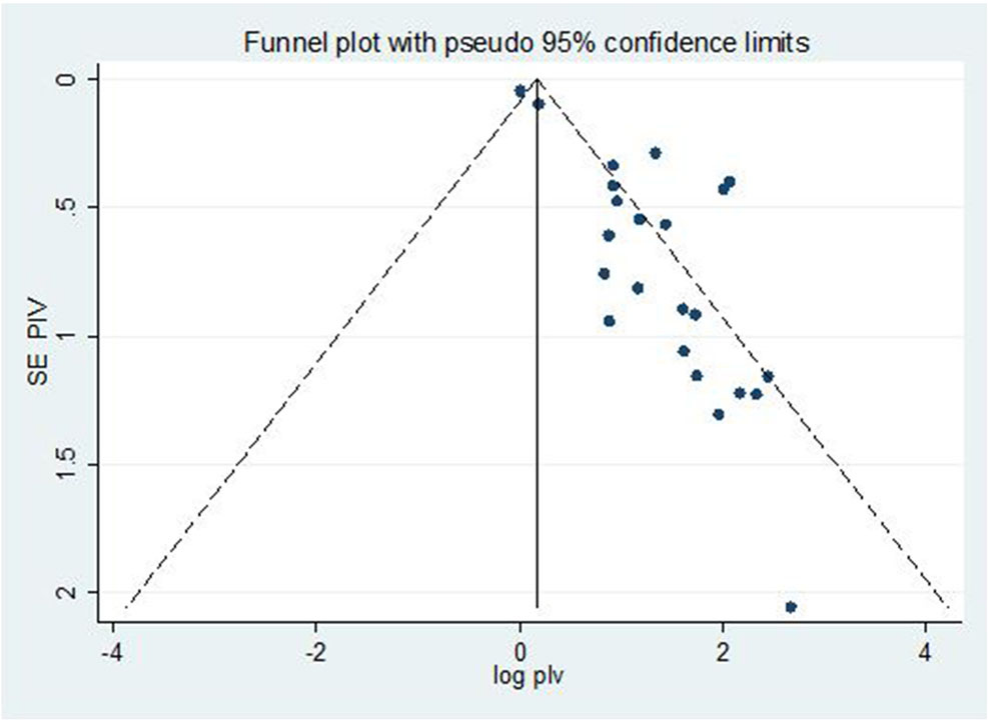
Meta funnel presentation of the prevalence of undiagnosed diabetes among adult individuals, 2007–2020, Africa. SE PIV = SQRT (prevalence*(100-prevalence)/sample size), log plv = LN of prevalence

**Fig. 3:**
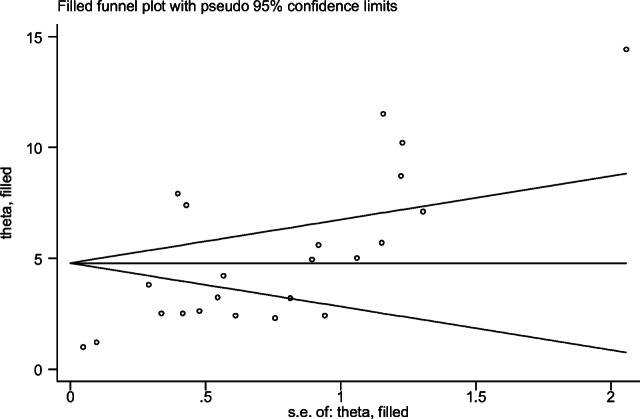
Filled funnel plot presentation of the prevalence of undiagnosed diabetes among adult individuals, 2007–2020, Africa

Both funnel plots of precision asymmetry and the Egger’s test of the intercept indicated the presence of publication bias in the studies. Visual examination of the funnel plot showed it to be asymmetric (Fig. 2), and Egger’s test of the intercept (B0) was 2.3 (95% CI: 1.63–3.04 *p* < 0.05). To mitigate against publication bias, we applied a trim and fill analysis in the random effects model. It ascertained that there are missed studies for publication (Fig. 3). According to this finding the result of trim and fill analysis was different from the first result.

### Prevalence and distribution of undiagnosed diabetes mellitus among adult individuals in Africa

**Fig. 4:**
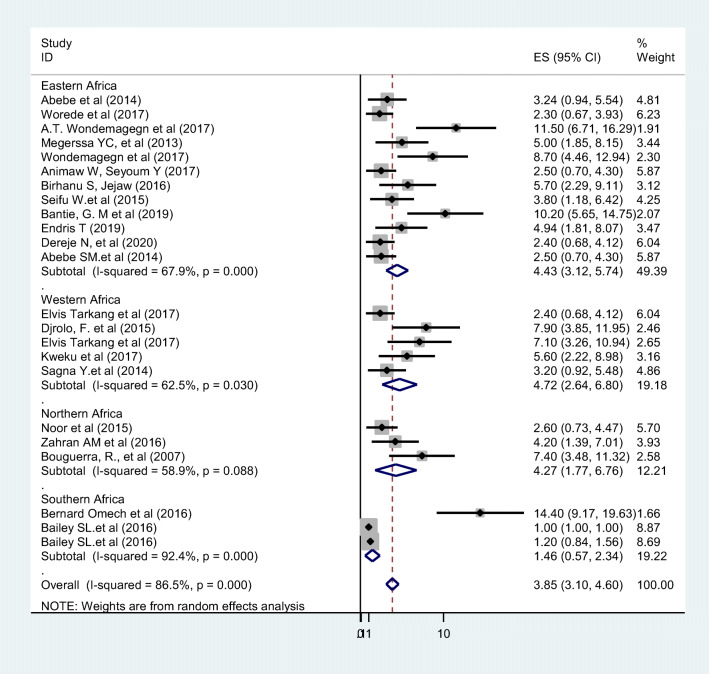
Forest plot of 23 studies assessing prevalence of undiagnosed diabetes among adult individuals, 2007–2020, Africa

The prevalence of undiagnosed diabetes ranges from 1.001% in a Zambia study [46] to 14.4%, reported in study conducted in Botswana [43]. Because the I^2^ static test for heterogeneity indicated significant difference between the studies (I^2^ = 86.4%, *p* < 0.05) and because theoretically we expected that the study settings and socio-economic contexts might differ radically across these studies, we fitted a DerSimonian and Laird random effect model to estimate the pooled prevalence of undiagnosed diabetes [49, 50]. In the model each individual study is given a weight based on its reported effect size and sample size [51]. The studies with the largest weight were Bailey.et al. [46], Elvis Tarkang et al. [40] and Worede et al. [34] with respective weight of 8.87%, 6.23% and 6.04% respectively. Smaller weights were given for Omech et al. 1.66% [43], and A.T.Wondemagegn et al. 1.91% [33]. The average pooled estimate of undiagnosed diabetes among adult population was 3.85 (95% CI: 3.10–4.60) (Fig. 4). Sub-group analysis by geographic region found that the pooled prevalence of undiagnosed diabetes in Eastern Africa was 4.43 (95% CI: 3.12–5.74), in Western Africa 4.72 (95% CI: 2.64–6.80), Northern Africa 4.27 (95% CI: 1.77–6.76) and 1.46 (95%CI: 0.57–2.34) in Southern Africa (Fig. 4).

In spite of sub-group analysis, the result still showed that the presence of heterogeneity across the studies was significant. Therefore, we performed meta regression analysis using publication year, sample size and country as a covariate. The result showed the listed covariates were not significant for the presence of heterogeneity across the studies (Table 2). Observed heterogeneity can be presumed to indicate real patterns or could reflect possible within-country heterogeneity.

**Table 2:** Meta regression results on selected variables in studies conducted from 2007 to 2020, Africa

Covariate		Coefficient	*P* value
Publication years		−0.41	0.46
Sample size		−0.001	0.134
Country	Benin	6.7	0.6
	Botswana	13.4	0.32
	Burkina Faso	2.2	0.87
	Egypt	3.2	0.81
	Ethiopia	4.3	0.66
	Ghana	11.7	0.27
	South Africa	0.2	0.99
	Sudan	1.6	0.90
	Tunisia	6.4	0.62

## Discussion

This systematic review and meta-analysis attempted to estimate the pooled prevalence of undiagnosed diabetes among adult individuals in Africa and its sub-regions. We found high prevalence of undiagnosed diabetes (3.85%) among African adult population. This current study also found regional variation in the prevalence of undiagnosed cases, with the highest prevalence reported in Western Africa (4.72) followed by East Africa (4.43%) and North Africa (4.27%), and the lowest in Southern Africa (1.46%) respectively.

This finding is inconsistent with prevalence of UDM which was found 69.2% in Africa reported by the International Diabetes Federation (IDF) in 2017 of global estimates of diabetes prevalence [4, 52] and 40% in sub-Saharan Africa [13]. These discrepancies might be explained by the difference in availability of diagnostic modalities in the current study period [53, 54]. Individuals included in the current study may have better accessibility to screening services than individuals who participated in previous, much older studies [55].

Our results are nearly consistent with the most recent meta-analysis conducted in Africa, published on the Journal of Diabetes Research in 2019, which summarized and pooled the results of community-based studies to provide a continental level estimate of the undiagnosed diabetes mellitus [15]. They found that the prevalence for pooled UDM for the African population was at 5.37%. The study further assessed differences in the prevalence of UDM between rural and urban areas, and between two diagnostic methods (Oral Glucose Tolerance Test and Fasting Blood Glucose, the prevalence of the latter, 4.54% which matches our findings). Our study compliments these results by offering an extra insight into regional differences of this outcome.

Evidence showed that Africa was the greatest contributor to the global burden of disease attributed to undiagnosed diabetes. A comparison of our findings to the global estimates presented by the IDF indicate a decreasing trend in undiagnosed cases of the disease [56]. However, the overall pooled prevalence of UDM still remains critically high for public health concern. Globally, the finding of this systematic review is consistent with results from a large population-based nationwide survey conducted in China with UDM of 4.2% [57] and it was 4% in U.S [58, 59]. This lag in the global scale correlates with economic and developmental positionality of a country. More developed countries like the US and China boast a national surveillance that captures undiagnosed diabetes; tracking key risk factors, such as levels of glycaemia and lipids; and surveillance of high or emerging-risk populations such as racial and ethnic groups, children and youth, and those with prediabetes [60]. This may help in early identification of diabetes, which results in low prevalence of undiagnosed diabetes. There is also a disproportionate socioeconomic difference between Africa, China and the US particularly in terms of availability and access to health services.

Another crucial finding from this study was the considerable variation in the prevalence of diabetes across different regions of Africa. While the prevalence of undiagnosed diabetes in North Africa and East Africa was consistent with the average pooled prevalence for whole of Africa, there was a significant slit difference with West Africa and Southern Africa, at 4.72% and 1.46% respectively. The high prevalence of undiagnosed diabetes in West Africa could be explained by the fact that majority of studies which were included were from Ghana where a high obesity burden was reported during the study period [61],which is the primary risk factor for diabetes [31, 62]. The finding from Southern Africa shows a relatively lower prevalence of undiagnosed diabetes when compared to other regions. One explanation for the low estimate might be the high rate of urbanization [63], and improved access to health services [64]. Urbanization can improve the ease of accessing the healthcare and as a result, low prevalence of undiagnosed diabetes in the region.

The differences in regional estimates may further be explained by the lack of variability and representation in the studies that were included in the meta-analysis. The estimated prevalence from this study rely on both the availability and quality of data used. Only 10 countries were represented in this study, with a positively skewed number of studies coming from Ethiopia and Ghana.

This study also brought to our attention the considerable lack of population-based studies on the prevalence of diabetes from French-speaking regions of Africa. Only 2 out of the 23 studies included in the systematic review and meta-analysis came from Francophone countries. This could be due to methodological bias in our inclusion criteria that only permitted studies done in English. This discrepancy may not be factored in our estimates for the pooled prevalence of undiagnosed diabetes among adults in Africa.

Delay in diagnosing diabetes can increase the risk of both long and short-term complications of the disease to patients. Risk factors for micro and macrovascular complications in undiagnosed diabetes are very common and are as frequent as in diagnosed diabetes. In addition to an increased risk of hyperglycemia, the patient with undiagnosed diabetes is more prone to hypertension, hypercholesterolemia, LDL cholesterol, hypertriglyceridemia and obesity [58, 65].

Early detection and treatment can improve the outlook for people with Type 2 Diabetes and other chronic diseases, since timely control decreases the risk of complications [66, 67]. Timely diagnosis and treatment can also minimize the overall expenditure of diabetes as well as prevent further hyperglycemia related cardiovascular diseases [68]. It is important to bear in mind that currently half of patients with Type 2 Diabetes already have some evidence of complications at the time of diagnosis. Patients self-management training has been considered an important part of clinical management to prevent diabetes-related debilitating complications. Over the years, educational techniques have evolved, and these have shifted from didactic presentations to interventions involving patient empowerment [69, 70]. For instance, in a recent study it was reported that diabetic subjects that acknowledge the HbA1c target and self-monitoring blood sugar were more commonly have well regulated type 2 diabetes mellitus compared to those not [71]. Not only in diabetes mellitus but also in other chronic diseases, such as hypertension, education of the subjects improves disease control and diagnosis. It was noted in another study that patients with hypertension who were aware of the normal blood pressure range and treatment targets have more common well controlled hypertension [72].When patients remain undiagnosed with diabetes, they miss out on the advantages of an early treatment regimen. Results from this study suggest that there is need for further action to increase efforts for early diagnoses and identification of diabetes in the areas where undiagnosed diabetes is still high within the continent.

## Study limitations

We only included studies from peer-reviewed English-language journals, which may have restricted our findings. Though searching was done for unpublished papers, only published studies were included. Different scholars used different calibration methods to estimate blood glucose level, which may also introduce variations across studies. The lack of variability and even representation in the studies that were included in the meta-analysis means that some people with the disease could have been missed. The findings of this study would be best interpreted by considering these analytical limitations and the limitations of the original studies in mind.

## Conclusion

This meta-analysis found that the prevalence of undiagnosed diabetes mellitus among adult individuals in Africa was high, and of consistent magnitude with that reported in other geographic regions. Given the high overall prevalence of undiagnosed diabetes in Africa, ministries of health and other non-profitable health-related organizations should pay more attention to scaling health education and awareness creation regarding early identification and screening for diabetes. Future research in Africa should focus on identifying appropriate strategies to increase early detection of diabetes.

## Electronic supplementary material

ESM 1 (PDF 644 kb)

ESM 2 (PDF 524 kb)

ESM 3 (DOC 69 kb)

## Data Availability

The data supporting the findings described in this article are provided.

## Abbreviations

CI: Confidence interval
DM: Diabetes Mellitus
IDF: International Diabetes Federation
NOS: Newcastle-Ottawa Scale
OR: Odds ratio
UDM: Undiagnosed Diabetes Mellitus
WHO: World Health Organization
