# Supplementary material for: A systematic analysis on prevalence and sub-regional distribution of undiagnosed diabetes mellitus among adults in African countries
Source: J Diabetes Metab Disord. 2020 Sep 22;19(2):1931–41. doi: 10.1007/s40200-020-00635-9 (PMC7843872; doi:10.1007/s40200-020-00635-9)
Supplement: Supplementary file 1 — (PDF 644 kb) [file 40200_2020_635_MOESM1_ESM.pdf]

Databases	Search Terms1438	No of articles identified
**PubMed**	("epidemiology"[Subheading] OR "epidemiology"[All Fields] OR "prevalence"[All Fields] OR "prevalence"[MeSH Terms]) AND undiagnosed[All Fields]) AND ("diabetes mellitus"[MeSH Terms] OR ("diabetes"[All Fields] AND "mellitus"[All Fields]) OR "diabetes mellitus"[All Fields])) OR ("diabetes mellitus"[MeSH Terms] OR ("diabetes"[All Fields] AND "mellitus"[All Fields]) OR "diabetes mellitus"[All Fields] OR "diabetes"[All Fields] OR "diabetes insipidus"[MeSH Terms] OR ("diabetes"[All Fields] AND "insipidus"[All Fields]) OR "diabetes insipidus"[All Fields])) AND mellitus[All Fields]) AND ("adult"[MeSH Terms] OR "adult"[All Fields])) AND ("population"[MeSH Terms] OR "population"[All Fields] OR "population groups"[MeSH Terms] OR ("population"[All Fields] AND "groups"[All Fields]) OR "population groups"[All Fields])) AND ("africa"[MeSH Terms] OR "africa"[All Fields])	1187
**CINAHL (Plus with full text)**	**Thesaurus terms:** Prevalence, undiagnosed, diabetes mellitus, adult, population, Africa<br>**Search Terms:** " Prevalence *" OR Epidemiology * OR " burden *" and " undiagnosed *" OR " Hiden " AND " diabetes mellitus "OR" diabetes " AND mellitus AND "Africa<br>(S1): filter: English, peer reviewed, exclude Medline<br>(S2): with additional filter: Africa<br>S1 OR S2	33
**Cochrane Library**	**MeSH terms:** Prevalence (MeSH), undiagnosed (MeSH), diabetes mellitus (MeSH), adult (MeSH), population (MeSH), Africa (MeSH).<br>**Search Terms:** (Prevalence * OR Epidemiology * AND undiagnosed * AND (diabetes mellitus [MeSH] OR diabetes [MeSH] AND mellitus *AND Adult *AND population *AND Africa AND English [la]	19
**Embase**	**Emtree terms:** Prevalence, undiagnosed, diabetes mellitus, adult, population, Africa	23

	**Search 1 Terms:** (Prevalence * OR epidimeology *AND undiagnosed * AND (diabetes mellitus * OR diabetes *AND mellitus *AND Adult *AND population *AND Africa (embase) NOT (medline)<br>**Search 2 Terms:**<br>(Prevalence NEXT/1 epidemiology * AND undiagnosed * NEXT/1 Hidden * AND diabetes mellitus NEXT/1 phone* AND (diabetes OR mellitus) AND (Adult) AND population and Africa AND english:la AND [embase]/lim NOT [medline]/lim<br>filter English only and excluded Medline	
**Others websites and Grey Literature; Google and Google Scholar**	Variety of key terms used from above searches	176
