# Supplementary material for: A systematic analysis on prevalence and sub-regional distribution of undiagnosed diabetes mellitus among adults in African countries
Source: J Diabetes Metab Disord. 2020 Sep 22;19(2):1931–41. doi: 10.1007/s40200-020-00635-9 (PMC7843872; doi:10.1007/s40200-020-00635-9)
Supplement: Supplementary file 2 — (PDF 524 kb) [file 40200_2020_635_MOESM2_ESM.pdf]

Table S1: Risk of Bias assessment Tool of Eligible Articles by using the Hoy 2012 tool

NO	Study ID	Representa tion	Sampling	Random selection	Non response bias	Data collection	Case Definition	Reliability and validity of study tool	Method of data collection	Prevalence period	Numerator and denominator	Summary Assessment
1	Bouguerra, R., et al	Low risk	Low risk	High risk	Low risk	Low risk	Low risk	High risk	Low risk	low risk	Low risk	Medium risk
2	Megerssa YC, et al	High risk	Low risk	Low risk	High risk	Low risk	Low risk	Low risk	Low risk	Low risk	Low risk	Low risk
3	Abebe et al	Low risk	Low risk	High risk	High risk	Low risk	Low risk	High risk	Low risk	Low risk	Low risk	Medium risk
4	Sagna Y.et al	Low risk	Low risk	High risk	Low risk	High risk	High risk	High risk	Low risk	Low risk	Low risk	High risk
5	Seifu W.et al	Low risk	Low risk	Low risk	Low risk	Low risk	Low risk	Low risk	Low risk	Low risk	Low risk	Low risk
6	Noor et al	Low risk	Low risk	High risk	Low risk	Low risk	Low risk	Low risk	Low risk	Low risk	Low risk	Medium risk
7	Djrolo, F. et al	Low risk	High risk	High risk	High risk	High risk	High risk	High risk	High risk	High risk	High risk	High risk
8	Bailey SL.et al	High risk	High risk	High risk	High risk	High risk	High risk	High risk	High risk	High risk	High risk	High risk
9	Bailey SL.et al	High risk	High risk	Low risk	Low risk	Low risk	Low risk	Low risk	Low risk	Low risk	Low risk	Low risk
10	Birhanu S.et al	Low risk	Low risk	Low risk	Low risk	Low risk	Low risk	Low risk	Low risk	Low risk	Low risk	Low risk
11	Zahran AM et al	Low risk	Low risk	Low risk	Low risk	Low risk	High risk	Low risk	Low risk	Low risk	Low risk	Low risk
12	Bernard Omech et al	High risk	Low risk	Low risk	High risk	Low risk	Low risk	High risk	Low risk	Low risk	Low risk	High risk
13	Worede et al	High risk	Low risk	Low risk	High risk	Low risk	Low risk	Low risk	Low risk	Low risk	Low risk	Low risk
14	Alex Kojo Anderson	Low risk	Low risk	Low risk	Low risk	Low risk	Low risk	Low risk	Low risk	Low risk	Low risk	Low risk
15	Elvis Tarkang et al	Low risk	Low risk	Low risk	Low risk	Low risk	High risk	Low risk	Low risk	Low risk	Low risk	Low risk
16	Elvis Tarkang et al	Low risk	Low risk	Low risk	Low risk	Low risk	Low risk	Low risk	Low risk	Low risk	Low risk	Low risk
17	A.T. Wondemagegn et al	Low risk	Low risk	Low risk	Low risk	Low risk	Low risk	Low risk	Low risk	Low risk	Low risk	Low risk
18	Kweku et al	Low risk	Low risk	Low risk	Low risk	Low risk	Low risk	Low risk	Low risk	Low risk	Low risk	Low risk
19	Wondemagegn et al	Low risk	Low risk	Low risk	Low risk	Low risk	Low risk	Low risk	Low risk	Low risk	Low risk	Low risk
20	Animaw W, Seyoum Y	Low risk	Low risk	Low risk	Low risk	Low risk	Low risk	Low risk	Low risk	Low risk	Low risk	Low risk
21	Bantie, G. M et al	Low risk	Low risk	Low risk	Low risk	Low risk	Low risk	Low risk	Low risk	Low risk	Low risk	Low risk
22	Endris T	Low risk	Low risk	Low risk	Low risk	Low risk	Low risk	Low risk	Low risk	Low risk	Low risk	Low risk
23	Dereje N, et al	Low risk	Low risk	Low risk	Low risk	Low risk	Low risk	Low risk	Low risk	Low risk	Low risk	Low risk
24	Abebe SM.et al	Low risk	Low risk	Low risk	Low risk	Low risk	Low risk	Low risk	Low risk	Low risk	Low risk	Low risk

25

26 **Risk of bias assessment tool: Yes (low risk); No (high risk)**

27 1. Representation: Was the study population a close representation of the national population?

28 2. Sampling: Was the sampling frame a true or close representation of the target population?

29 3. Random selection: Was some form of random selection used to select the sample OR was a census undertaken?

30 4. Non-response bias: Was the likelihood of non-response bias minimal?

31 5. Data collection: Were data collected directly from the subjects?

32 6. Case definition: Was an acceptable case definition used in the study?

33 7. Reliability and validity of study tool: Was the study instrument that measured the parameter of interest show to have reliability and validity?

34 8. Data collection: Was the same mode of data collection used for all subjects?

35 9. Prevalence period: Was the length of the prevalence period for the parameter of interest appropriate?

36 10. Numerators and denominators: Were the numerator(s) and denominator(s) for the parameter of interest appropriate?

The overall risk of bias scored based on the number of high risk of bias per study: low risk (≤2), moderate risk (3–4), and high risk (≥5).
